# Field Validation of a Non-carcinogenic and Eco-Friendly Disinfectant in a Stand-In Footbath for Treatment of Footrot Associated With *aprV2*-Positive Strains of *Dichelobacter nodosus* in Swiss Sheep Flocks

**DOI:** 10.3389/fvets.2022.812638

**Published:** 2022-06-13

**Authors:** Robin Michael Schmid, Adrian Steiner, Jens Becker, Sandra Baumberger, Salome Dürr, Maher Alsaaod

**Affiliations:** ^1^Clinic for Ruminants, Department of Clinical Veterinary Science, Vetsuisse-Faculty, University of Bern, Bern, Switzerland; ^2^Veterinary Public Health Institute, Department of Clinical Research and Veterinary Public Health, Vetsuisse-Faculty, University of Bern, Bern, Switzerland

**Keywords:** *Dichelobacter nodosus*, footrot, footbath, prewash waterbath, sheep

## Abstract

A national control program for virulent footrot is currently planned in Switzerland. Since commonly used disinfectants either contain heavy metals or are carcinogenic, the aim of this study was to verify the effectiveness of an eco-friendly and non-carcinogenic candidate disinfectant against *aprV2*-positive strains of *Dichelobacter nodosus*. Additionally, the effect of the selective use of long-acting oxytetracyclines was evaluated. A total of 18 farms with confirmed footrot infection, randomly allocated to two treatment groups: (1) with antibiotics (AB; *n* = 9) and, (2) no antibiotics (NAB; *n* = 9), were included. Claws were carefully trimmed and scored using a scale from 0 (clinically healthy) to 5 (complete loss of the horn capsule) and a prewash waterbath was implemented on 11 farms. Twice-weekly, repeated whole-flock stand-in footbaths with the candidate disinfectant (6%) were performed. Additionally, animals of group AB with a score ≥ 3 were administered oxytetracyclines by injection. On all farms, 10 days after last treatment, *aprV2*-positive strains could not be detected by risk-based sampling for real-time PCR analysis after 7–21 (median = 12) footbaths with a minimal culling rate of non-responders on nine farms. Farms without contact to other sheep remained without clinical signs of footrot for a minimum of 245 days (mean ± standard deviation: 293.6 ± 23.6). Antibiotic treatment did not reduce the number of footbaths needed. In contrast, a mean of 3.3 disinfecting footbaths could be saved by implementing a prewash waterbath. At animal level, individual and selective use of oxytetracyclines lead to a higher chance (odds ratio = 9.95; 95% CI: 3.54–27.95; *p* < 0.001) for a lesion score ≥ 3 to improve to a lesion score < 3 within 2 weeks compared to treatment without antibiotics. The test disinfectant is an effective and eco-friendly alternative for the planned Swiss footrot control program and selective use of oxytetracycline has a beneficial impact on the recovery of animals with lesion scores ≥ 3.

## Introduction

Footrot is a common cause of lameness in sheep (1) and has been described worldwide as an endemic disease (2, 3) with flock level prevalence ranging from 16.9% in Switzerland to 81.6% in the UK (4, 5). It has a negative impact on animal welfare (6, 7) and leads to economic losses due to a prolonged fattening period and reduced fertility due to an inadequate body condition (7–10).

In Switzerland, footrot-attributable costs for the years 2014–2030 without nationwide control measures have been estimated at 172'300'000 Swiss Francs (CHF^1^) (11). Hence, the Swiss Health Service for Small Ruminants (BGK) has established a protocol for footrot control in flocks (12) and two cantons (largest political unit of Switzerland) for an area-wide control. In 2024, a nationwide control program is planned to be initiated with the aim to reduce the prevalence of flocks harboring sheep that carry *aprV2*-positive strains of *Dichelobacter (D.) nodosus* to 1% within 5 years.^2^

*D. nodosus* is the etiological agent of footrot (13) and can be categorized as either benign or virulent according to the expression of the *aprB2* (thermolabile protease) and *aprV2* (thermostable protease) gene, respectively (14, 15). Environmental conditions and host susceptibility modulate the severity of clinical signs expressed by sheep harboring virulent and non-virulent strains of *D. nodosus* (16–19). On animal level, benign strains lead to mild interdigital dermatitis. The clinical signs of an infection with virulent strains vary from absence of symptoms (healthy carriers) to the characteristic underrunning of the hoof capsule culminating in the complete separation from the underlying epidermal tissue (15, 20). In rare cases, benign strains may also cause clinical signs of footrot (21–23), however, the risk of severe footrot lesions was significantly increased if virulent strains were present compared to the presence of benign strains (21).

A competitive real-time PCR (rtPCR) method has been established for the identification and classification of *D. nodosus* isolates targeting the protease genes (24). The rtPCR is not infallible and clinical scoring of all sheep prior to sampling is still necessary (25). But as the aim of the Swiss control program is the reduction of the prevalence of *aprV2*-positive flocks, this non-invasive and fast method is essential. Due to its high sensitivity, even clinically healthy carriers can be identified (25).

Various approaches have been established to control footrot. While vaccines have only led to a decrease of the in-flock prevalence (26–28), whole-flock treatments with macrolides have completely eliminated the virulent strain of *D. nodosus* in some flocks (29–31), but elimination was incomplete in another study (32). However, the veterinary use of antibiotics should be restricted to the lowest justifiable level. In Switzerland, macrolides, rated highest priority critically important antibiotics (33), are not licensed for use in sheep.^3^ Furthermore, the Swiss strategy on antibiotic resistance (StAR) provides guidelines, which prohibit a whole-flock treatment (34). The licensed and less critical oxytetracyclines have shown comparable effectiveness (35) and combined with a footbath, the cure rate was superior to that of disinfecting footbaths alone (36).

Alternatively, Greber et al. (25) successfully eliminated virulent strains of *D. nodosus* from 28 sheep flocks by careful claw trimming, weekly stand-in disinfecting footbaths (10% zinc sulfate) and minimal culling rate of non-responders without using antibiotics. To date, frequently used disinfectants in footbaths are zinc sulfate (ZnSO4; 10%), copper sulfate (CuSO4; 5%) and formaldehyde (4%) (25, 37–39). The latter is carcinogenic (40) whereas the others contain heavy metals and their disposal is challenging (41). Therefore, there is a need for alternative disinfectants, which must neither contain heavy metals nor formaldehyde and must be effective against *D. nodosus* and readily available as concentrate. The candidate disinfectant—composed of organic acids and glutaraldehyde—fulfilled all the criteria and displayed promising results in *in vitro* and *ex vivo* experiments at a concentration of 6% (42).

The aims of the present study were (i) to evaluate the effectiveness of the candidate disinfectant for treatment of footrot associated with virulent strains of *D. nodosus* strains using an adapted treatment protocol from Greber et al. (25), (ii) to identify factors being associated with the number of footbaths needed to reach *aprV2*-negative flock status confirmed by risk-based sampling for rtPCR analysis, and (iii) to investigate the effect of the selective use of long-acting oxytetracyclines.

## Materials and Methods

### Recruitment of Farms and Inclusion Criteria

A short information communication about the study design was distributed to all members of the BGK, representing about 26% of Swiss sheep farmers, via their newsletter magazine and published in two agricultural newspapers (*Schweizer Bauer*, October 2020; *Bauernzeitung*, January 2021). Interested farmers contacted the main investigator (RS) and were included if (i) a maximum of 100 sheep (including ewes, rams and lambs) were present at the first farm visit, (ii) at least one sheep was diagnosed with an advanced footrot lesion score ≥ 3 [according to the BGK adapted from Egerton and Roberts (43); Table 1], and (iii) the presence of the virulent strain was confirmed by rtPCR (24). Farmers volunteered to participate and provided informed written consent to strictly follow the study guidelines. If treatment protocol was not strictly followed, farms were immediately excluded.

**Table 1:** Footrot scoring system according to BGK^a^; adapted from Egerton and Roberts (43).

**Footrot scoring**	
Score 0	Healthy claw
Score 1	Mild interdigital dermatitis
Score 2	Extensive interdigital dermatitis with involvement of the axial horn
Score 3	Severe interdigital dermatitis and under-running of the horn of the heel and sole
Score 4	Further under-running spread to the abaxial walls of the hoof
Score 5	Loss of the horn capsule

a *BGK: https://www.xn–kleinwiederkuer-clb.ch/fileadmin/04_kleinwiederkaeuer/02_Programme_Projekte/Moderhinkeprogramm/Merkblatt_6_Moderhinke_deutsch.pdf (accessed December 9, 2019)*.

### Treatment Protocol

**Figure 1:**
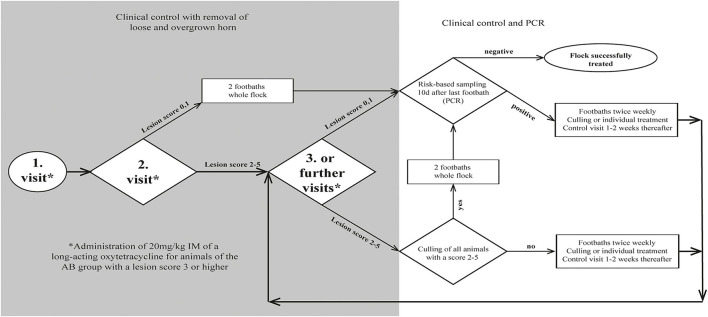
Flow chart for decision making during treatment of 18 sheep flocks with an *aprV2-*positive footrot status.

The study was carried out from September 2020 to May 2021. All visits were performed by the main investigator and conducted at week 0, 2, 4. Further visits were carried out at weekly to biweekly intervals until the complete absence of clinical signs of footrot (all sheep with a score <2; Figure 1). Recruited farms were randomly allocated to either the treatment group AB (antibiotic treatment; *n* = 9) or group NAB (no antibiotic treatment; *n* = 9). Not all farms entered the study at the same time, and to reduce seasonal effects, farms were enrolled as pairs into the study. From this pair, the allocation to the group was done by drawing a lot from a pot containing one lot of each treatment group. Excluded farms were replaced by enrolling new farms into the corresponding treatment group.

To allow the disinfectant solution to reach the infected tissue, overgrown, loose and underrun horn was carefully trimmed, taking care not to cause bleeding. Claws were scored (Table 1) by the main investigator, and classified as clinically affected when scored ≥ 2. To keep the number of sheep being treated with antibiotics as low as possible (prudent use of antibiotics), only individuals of group AB diagnosed with a score ≥ 3 were additionally treated with one dose of a long-acting oxytetracycline intramuscularly (20 mg/kg, IM). Farmers of both groups were recommended to treat lame animals additionally with a non-steroidal anti-inflammatory drug (NSAID) (Figure 1). Analgesic treatment was not part of the study protocol and was provided by the private veterinarian. Farmers decided independently which animals to treat after the first foot bath and used a variety of NSAIDs (ketoprofen *n* = 2, meloxicam *n* = 4, tolfenamic acid *n* = 1) at different dosages.

Twice a week, sheep of both groups were footbathed using a 6% solution of the candidate disinfectant (Desintec^®^ Hoofcare Special D, Dr. E. Graeub AG; containing 10% acetic acid, 8.8% glycolic acid, 6% glutaraldehyde) at a minimum liquid level of 6 cm to cover the coronary band for 10 min. The disinfecting solution was freshly prepared for each footbath. Prior to footbathing, cleaning of the claws was always mandatory. This was achieved by (i) manual removal of dirt, (ii) individual rinsing with a water hose, (iii) prewash walk-through/stand-in waterbath, or combinations thereof. Prior to footbath 1, a prewash waterbath was available on four farms. Prior to footbath 2, it was available on a further seven farms. Once available, it was used for all subsequent footbaths.

After each footbath, sheep were first confined to a clean concrete floor for at least 1 h. Afterwards, they were kept in a clean, dry and at least freshly bedded barn or on a pasture that had not been used by sheep for at least 4 weeks. In order to reduce contamination, farmers were advised to muck out and disinfect the stable after the first footbath. All removed claw material was disposed of via the household waste, and the claw trimming area was disinfected with the candidate disinfectant (6%). Foot shears were disinfected as described below.

### Farm Visit 1 (Week 0)

After claw trimming by an experienced claw trimmer from the BGK, all claws of each sheep were examined by the main investigator. No special measures regarding disinfection of the claw trimming tools were applied at this point. Each individual foot was scored, while the clinical score of each sheep was determined by the maximal individual foot score. If footrot lesions were present, underrun horn was carefully removed without damaging the subdermal tissue, and clinically affected sheep were marked for follow-up control. Other claw pathologies (e.g., claw horn disorders, deformation or poor claw quality) were documented and considered as risk factors for an infection with *D. nodosus* (44).

In order to confirm the presence of *D. nodosus* carrying the *aprV2* gene, a maximum of five 4-feet swab samples of animals with clinical signs were collected per farm, pooled according to Greber et al. (44) and analyzed as one sample. Animals with a score ≥ 3 of group AB were administered oxytetracycline, and the whole flock underwent first footbathing.

### Farm Visit 2 (Week 2)

At follow-up examination, all color-marked and newly lame sheep were scored and carefully trimmed by the main investigator, if obviously loose horn was identified. Disposable gloves were changed and used trimming tools were first cleaned with an alcohol-containing disposable towel (Eutertuch Agraro, Landi) and then disinfected with the candidate disinfectant (6%) according to a previously described protocol after each sheep (45). Sheep of group AB diagnosed with a score ≥ 3 were treated with oxytetracycline and sheep of both groups underwent the fifth footbath.

### Farm Visit 3 (Week 4), Further Control Visits and Follow-Up Evaluation

All animals were re-examined, scored and treated if necessary, as per farm visit 2. Farmers were recommended to either cull non-responders or initiate individual topical treatment by either spraying the candidate disinfectant (6%) onto the lesion by a disperser or applying it into the interdigital space via a soaked swab for at least 1 min. The swab was removed prior to footbathing (Figure 1).

As soon as clinical healing (score < 2) occurred for the whole flock, footbathing was discontinued and a risk-based sampling was performed according to the BGK eradication protocol after a waiting period of ≥ 10 days to allow the within-flock prevalence of *aprV2*-positive sheep to rise ≥ 20% in case of non-elimination (12). According to Greber et al. (44), a maximum of 30 animals were sampled and analyzed in pools-of-10. Sheep with a high (e.g., lame animals), moderate (e.g., rams or sheep with previous claw problems) or low (randomly selected animals) risk were sampled and allocated to pool one, two and three, respectively. Animals from a lower risk level were assigned to a higher risk level pool if necessary (Supplementary Figure 1). The number of sheep to be tested was determined according to the flock size with an assumed within-flock prevalence of 20% and a sensitivity and specificity of 90 and 98%, respectively (44). Treatment was considered successful, if the flock tested negative for the *aprV2* gene. Follow-up information on the clinical progress of the flock was obtained by telephone conversation with the farmers at 245–436 days after last sampling.

### Sampling, Pooling, and Laboratory Analyses

The feet were sampled with sterile dry cotton swabs (2 × 15 mm) by rubbing the swab on the outer rim of the lesion or in the interdigital cleft if no lesion was present. According to a standardized protocol by Locher et al. (46), one clean quarter of the same swab was used for each foot to obtain a 4-feet sample. To avoid cross-contamination between sheep, disposable gloves were changed after every sheep.

After sampling, the swabs were immediately placed into a 1.5 mL SC micro tube or 96-deep well-plate which contained 1 mL SV-lysis buffer (4 M guanidine thiocyanate, 0.01 M Tris–HCl, 1% β-mercaptoethanol) for at least 2 min. To prevent cross contamination, only every second well was filled, and the plates were locked with a silicon cover. Collected samples were transported at room temperature to the laboratory the same day (within 15 h) and stored at 4°C for a maximum of 1 week. After pooling according to Greber et al. (44), rtPCR-analyses were run in a single laboratory (Institute of Veterinary Bacteriology, University of Bern) and rated positive at a threshold cycle (Ct) < 40 (24). In order to validate each rtPCR assay, a negative (H_2_O) and positive control sample was analyzed alongside.

### Sample Size Calculation and Statistical Analysis

The sample size was calculated using a free online-tool (Epitools, Ausvet),^4^ to detect a difference of at least four footbaths between groups AB and NAB. With the assumption of an average of 12 and 16 footbaths for the two groups, respectively, and an expected variance of nine footbaths, a sample size of 18 flocks was calculated with confidence level set at 95% and power at 80%.

Descriptive statistics, equal-variance *t*-tests to detect differences between the two treatment groups in initial flock size, within-flock prevalence of clinical footrot and advanced footrot lesions, and the survival plots were carried out with the software package NCSS^2020^ (NCSS LLC). Further analyses were performed with the statistical software R.^5^ Univariable analyses were performed and variables were only offered to the multivariable models if *p* < 0.25. Firstly, factors associated with individual animal recovery from score ≥ 3 to score < 3 were examined using a generalized linear mixed-effect model using logit function with the farm fitted as a random effect. From the final model output, intraclass correlation coefficients (ICC) were calculated from covariance parameter estimates using the formula:

ICC = var(farm)/(var(farm) + var(residual))

where var(farm) is the covariance parameter estimate of the random effect farm, and var(residual) is the residual covariance parameter estimate. Explanatory variables at farm, management and animal level were analyzed as fixed effects (Supplementary Table 1). A second set of analyses were run to identify factors associated with the number of footbaths needed to eliminate the disease from the flocks. Two generalized linear models with a continuous and binary outcome, respectively, were built. In the first model, the number of footbaths as outcome variable was modeled using poisson regression. In the second model, a binomial approach with the threshold set at the median number of footbaths needed to completely eliminate footrot on the farm (*n* = 12) was performed. Potential risk factors are presented in Table 2. The models were optimized by stepwise backward elimination of non-significant parameters and Akaike's Information Criterion (AIC) was used to choose the best model. The level for significance was set at *p* = 0.05 and for tendency, *p*-values between 0.05 and 0.09 were considered.

**Table 2:** Definition of factors potentially affecting the number of footbaths needed for complete elimination of virulent footrot in Swiss sheep flocks.

**Potential model variables**	**Statistical summary (*n* = 18 farms)**	**Group NAB (*n* = 9)**	**Group AB (*n* = 9)**
**FLOCK FACTORS (CONTINUOUS)**			
Flock size	9–67 (median, 28)	9–67 (median, 21)	14–52 (median, 38)
Prevalence of footrot^a^	23–93% (median, 49.5%)	23–62% (median, 43.0%)	31–93% (median, 68.0%)
Prevalence of advanced lesions^b^	11–71% (median, 30.5%)	11–50% (median, 29.0%)	21–71% (median, 32.0%)
Prevalence of other claw pathologies	8–64% (median, 29.0%)	12–43% (median, 30.0%)	8–64% (median, 28.0%)
Change in number of lambs^c^	−3 to 26 (median, 2)	0–26 (median, 5)	−3 to 26 (median, 1)
**FLOCK FACTORS (CATEGORICAL)**			
**Average sum score^d^**			
Low (<5.5)	6 (33.3%)	4 (44.4%)	2 (22.2%)
Medium (≥5.5, <7)	6 (33.3%)	3 (33.3%)	3 (33.3%)
High (≥7)	6 (33.3%)	2 (22.2%)	4 (44.4%)
**SWA^e^ present**			
Yes	3 (16.7%)	0 (0%)	3 (33.3%)
No	15 (83.3%)	9 (100.0%)	6 (66.7%)
**Treatment group**			
Antibiotic treatment^f^	9 (50.0%)	0 (0.0%)	9 (100.0%)
No antibiotic treatment	9 (50.0%)	9 (100.0%)	0 (0.0%)
**MANAGEMENT FACTORS**			
**Permanent access to a concrete outdoor paddock**			
Yes	7 (38.9%)	3 (33.3%)	4 (44.4%)
No	9 (50.0%)	5 (55.6%)	4 (44.4%)
Not applicable^g^	2 (11.1%)	1 (11.1%)	1 (11.1%)
**Prewash waterbath prior to first footbath**			
Yes	4 (22.2%)	2 (22.2%)	2 (22.2%)
No	14 (77.8%)	7 (77.8%)	7 (77.8%)
**Prewash waterbath prior to subsequent footbaths^h^**			
Yes	11 (61.1%)	6 (66.7%)	5 (55.6%)
No	7 (38.9%)	3 (33.3%)	4 (44.4%)
**Liming with calcium carbonate**			
Yes	7 (38.9%)	4 (44.4%)	3 (33.3%)
No	10 (55.6%)	4 (44.4%)	6 (66.6%)
NA^i^	1 (5.6%)	1 (11.1%)	0 (0.0%)
**Muck out and disinfecting at visit 1**			
Yes	10 (55.6%)	7 (77.8%)	3 (33.3%)
No	7 (38.9%)	1 (11.1%)	6 (66.6%)
NA^i^	1 (5.6%)	1 (11.1%)	0 (0.0%)
**OTHERS**			
**Season at the start of the treatment**			
September–November	4 (22.2%)	1 (11.1%)	3 (33.3%)
December–February	12 (66.7%)	6 (66.7%)	6 (66.6%)
March–May	2 (11.1%)	2 (22.2%)	0 (0.0%)
June–August	0 (0.0%)	0 (0.0%)	0 (0.0%)

a *Additionally categorized into three categories with approximately the same number of farms per category*.

b *Score ≥ 3 according to BGK*.

c *Difference in number of lambs between first and last visit*.

d *Average sum of the 4 individual foot scores of clinically affected sheep*.

e *SWA: Swiss white alpine sheep*.

f *One dose of a long-acting oxytetracycline (20 mg/kg, IM)*.

g *Not applicable: sheep were kept on pasture*.

h *On seven farms, the prewash waterbath was not available from the beginning or could not be used because the court was needed for claw trimming*.

i *NA: not available*.

## Results

### Farms

A total of 21 farms from eleven cantons were included in this study. Three farms were excluded because no lesion ≥ 3 was present (farm 3) or the treatment protocol was not strictly followed (farms 8 and 15).

Descriptive results are given in Table 2. No difference between groups AB and NAB was detected regarding flock size (*p* = 0.61) and the within-flock prevalence of advanced lesions (*p* = 0.20), but there was tendency (*p* = 0.07) of a higher within-flock prevalence of clinical footrot in group AB (median = 68%) as compared to group NAB (median = 43%).

### Improvement of Advanced Footrot Lesions

A total of 177 sheep were diagnosed with an advanced footrot lesion (score ≥ 3) at farm visit 1, of which 106 were treated with oxytetracyclines. The odds for an improvement of the overall score from score ≥ 3 to score < 3 after four footbaths (interval of 2 weeks) was higher with antibiotic treatment (odds ratio = 9.95; 95% CI: 3.54–27.95) and with a prewash waterbath prior to the first footbath (odds ratio = 13.40; 95% CI: 2.55–70.31; Table 3). In contrast, the use of NSAIDs had a negative impact on the healing process of advanced footrot lesions (odds ratio = 0.21; 95% CI: 0.07–0.65). The initial footrot score and the number of feet affected had no influence on the recovery of the advanced lesions. The adjusted and conditional ICC of the model was calculated at 0.045 and 0.025, respectively, indicating that the variance of healing from score ≥ 3 to <3 is not dependent on the flock when the fixed effects are considered.

**Table 3:** Factors affecting the improvement of the overall score from a score ≥ 3 to a score <3 within 2 weeks (= 4 footbaths) in individual sheep identified by multivariable mixed effect model.

	**Odds ratio (95% CI)**	***p*** **-value**
NSAID^a^	0.21 (0.07–0.65)	0.006
Water bath prior to the first footbath	13.40 (2.55–70.31)	0.001
Antibiotic treatment^b^	9.95 (3.54–27.95)	<0.001

a *NSAID: non-steroidal anti-inflammatory drug*.

b *One dose of a long-acting oxytetracycline (20 mg/kg, IM)*.

### Number of Footbaths Required for Footrot Elimination

**Figure 2:**
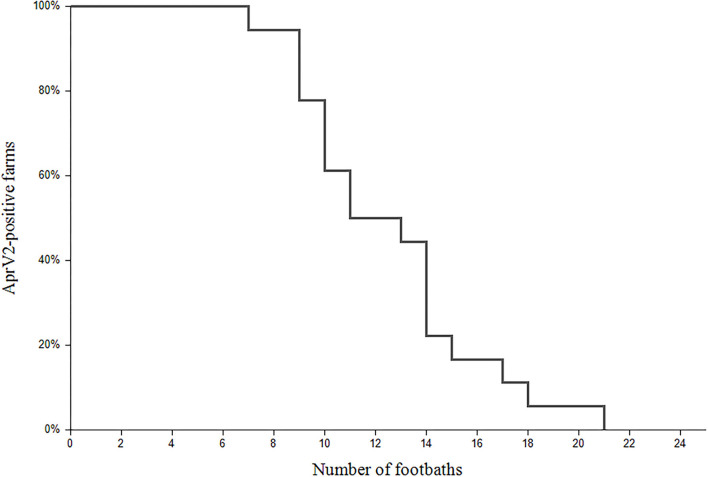
Survival plot of the *aprV2*-positive status during the treatment of footrot in 18 flocks. The *x*-axis refers to the number of footbaths and the *y*-axis represents the percentage of the farms (%) with *aprV2*-positive status.

All 18 farms were tested negative for the virulent strain of *D. nodosus* at the end of the study. The number of footbaths needed ranged from 7 to 21, while 50% of the farms were tested negative after 11 footbaths (Figure 2). After four additional footbaths, another six farms were tested negative. The remaining three (farm 9, 10, and 13) were tested negative after 17, 18, and 21 footbaths, respectively. On farm 9, there was only one sheep with clinical signs after 11 footbaths. This sheep was culled after footbath number 15 and the rest of the flock was tested negative after two additional footbaths. On farms 10 and 13, a positive *aprV2*-status was detected without observing clinical signs at the time of sampling (after 9 and 14 footbaths, respectively). On farm 10, clinical signs were observed 1 week after the sampling, whereas no obvious signs of footrot were evident on farm 13 until one sheep was severely lame 2 weeks later. Claw trimming performed by the farmer revealed an advanced footrot lesion and this animal was immediately isolated and culled. The rest of the flock was tested negative after two additional footbaths (*n* = 21). In contrast, two clinically affected animals were observed 10 days after the “last” footbath (*n* = 9) on farm 7. The treatment was continued and after culling the affected animals, the rest of the flock tested negative after 14 footbaths.

Ten of the 18 farmers did not report any clinical signs of footrot for at least 62–213 days (mean ± standard deviation: 114.7 ± 48.5), which reflected the period between last sampling (completion of the program) and the beginning of the following alpine summer pasturing. Seven flocks, in which neither alpine summer pasturing nor any contact to other sheep occurred, freedom from clinical signs of footrot was reported up to the day of the follow-up survey, i.e., for 245–325 days (293.6 ± 23.6). In the remaining farm, all sheep were sold 113 days after the last sampling not showing any clinical signs at that time.

**Figure 3:**
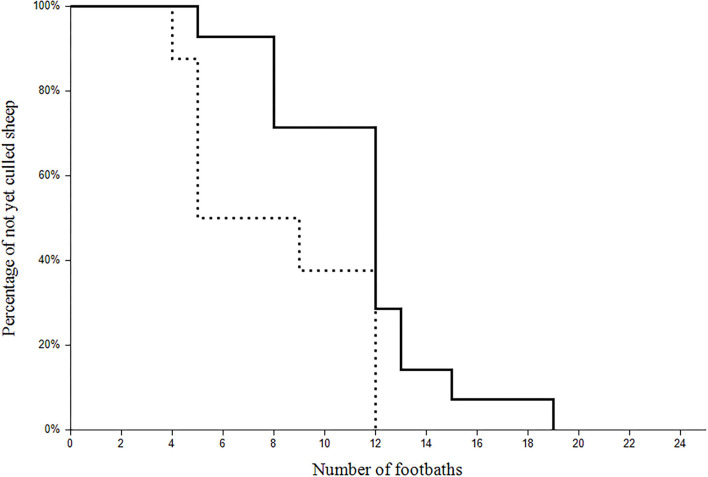
Survival plot of the of the 22 clinically affected sheep culled during the study. The *x*-axis refers to the number of the footbaths and the *y*-axis represents the percentage of the not yet culled sheep with (dotted line; *n* = 8) or without (continuous line; *n* = 14) a co-existing disease.

To reach the *aprV2*-negative status, 22 sheep were culled because of footrot on nine different farms. If sheep additionally suffered from another underlying disease (e.g., chronic mastitis; *n* = 8), they were culled after the second and third visit, respectively, whereas sheep suffering from footrot only (*n* = 14) were culled later (Figure 3).

The two types of regression analyses (continuous and binary outcomes) investigating the number of footbaths required to eliminate footrot in the farm revealed that the only consistent variable was “prewash waterbath”. In farms, on which the disinfecting footbath was preceded by a prewash waterbath (*n* = 11), a mean of 3.3 footbaths could be saved (Table 4).

**Table 4:** Mean numbers of footbaths required for complete elimination of virulent strains of *D. nodosus* at flock level with and without a prewash waterbath.

	**Number of footbaths (95% CI)**	***p*** **-value**
No prewash waterbath prior to disinfecting footbaths	14.6 (12.0–17.7)	
Prewash waterbath prior to disinfecting footbaths	11.3 (9.5–13.4)	0.055

## Discussion

Using our novel approach, no virulent strains of *D. nodosus* could be detected 10 days after the last footbath on all 18 farms included in this study. To the best of our knowledge, treatment of footrot with a non-carcinogenic and eco-friendly disinfectant has not been described to date. Compared to commonly used disinfectants, the number of footbaths needed was similar, even though the initial clinical prevalence was higher than in a previous study (25).

By increasing the weekly treatment from once to twice a week, a faster elimination of virulent footrot was achieved. Especially during the planned Swiss control program, where *aprV2*-positive flocks are subjected to transport and traveling restrictions this may be important for farmers. Additionally, up to three footbaths can be saved on farms with a prewash waterbath compared to farms without. It is an easily implementable intervention and to our knowledge, does not contribute to the development of antibiotic resistance. To our knowledge, similar results of using a prewash waterbath in sheep have not been described. In cattle, however, prewashing the claws with a waterbath is recommended (47), and it has been demonstrated that less organic matter was transferred into the disinfecting bath due to cleaner claws (48). The beneficial effect shown in the present study can be explained in two ways: first, the disinfectant solution gets in closer contact with the infected tissue when claws are clean (20, 49) and second, the degree of inhibition of the disinfectant solution by organic matter is lower after effective prewashing (50).

At flock level, elimination of virulent footrot was possible with or without antibiotic treatment, whereas the number of footbaths needed did not significantly differ between the two treatment groups. The tendency of a higher initial prevalence of footrot in group AB may have reduced the detection probability of finding a difference between the groups. In addition, antibiotics were only administered to severely affected individuals in the flock. Even though whole-flock treatments with antibiotics successfully eliminated virulent strains of *D. nodosus* (30, 31), blanket antibiotic treatment of all sheep of a flock is not in accordance with the StAR guidelines and therefore not applicable in Switzerland (34).

Disinfecting footbaths are not recommended anymore, as they only reduce superficial bacterial load and do not penetrate into the infected claw horn (51). In order to enable the disinfectant solution to reach the infected tissue, overgrown and obviously loose horn was carefully removed according to Greber et al. (25). Claw trimming restored a physiological hoof shape improving the gait of the sheep (52) and contributed to an accurate scoring of footrot lesions (1). Kaler et al. (53) reported slower healing of footrot lesions when trimmed rather than solely treated with antibiotics. This can be explained by excessive claw trimming which leads to damage of the pododermis and thus bleeding (5). Therefore, careful removal of underrun horn without causing any bleeding was anticipated in the current study. In addition, except from the use before the first footbath, foot shears were disinfected after individual sheep examination to reduce transmission of pathogens between sheep to the lowest possible level (45).

At animal level, the additional antibiotic treatment of individual animals in group AB had a beneficial effect on the healing process of advanced lesions in comparison with footbathing alone. This finding is in agreement with a previous study (53). With the higher chance of recovery after 2 weeks‘ treatment, animal welfare may be improved since the clinical footrot score is a good indicator for altered pain behavior (54). As well as oxytetracyclines (32, 35, 53) aminopenicillins (55) and a combination of penicillin and streptomycin (56) have also been shown to be effective against *D. nodosus* and may provide a more prudent alternative to macrolides (highest priority critically important antibiotics). Nevertheless, based on their categorization by the World Health Organization (33), we consider oxytetracyclines (rated highly important) as the preferable choice against *D. nodosus* as aminoglycosides and aminopenicillins are rated “critically important antibiotics”.

The initial footrot score and the number of feet affected had no effect on the healing process of advanced lesions. This contradicts previous findings (56, 57) but as our investigation only focused on an improvement from score ≥ 3 to score < 3 and not complete clinical healing, a comparison is difficult.

The culling of chronically infected sheep may reduce the treatment duration of flocks (17, 25). Even though such sheep represent a constant source of infection (38, 58), farmers are often unwilling to cull them (25). Likewise, farmers tended to be reluctant regarding culling, and most sheep were only excluded after the fourth visit and not as recommended after the third. Conversely, co-existing diseases seemed to facilitate the decision to cull an individual, and on average, those animals were sorted out at an earlier stage. Another motivational factor for culling individual animals could be the increased costs of the prolonged treatment, as the disinfecting solution needs to be renewed for every footbath. Additionally, governmental compensation payments could lead to faster elimination of infected sheep. Especially in small flocks, however, emotional and breeding related values may play a pivotal role for farmers not to cull their animals.

The risk-based sampling used for rtPCR diagnostics is a non-invasive, sensitive and cost-effective diagnostic tool, as not every animal needs to be tested and samples can be analyzed in pools-of-10 (44). Respecting a 10-day waiting period between the last footbath and sampling is part of the governmental Swiss footrot control program. In case clinically healthy carriers were present, they would transmit *D. nodosus* to other sheep during this waiting period revealing failure of the sanitation measures when applying diagnostics using risk-based sampling. In an experimental study, all sheep turned PCR positive (Ct < 40) after having had contact with an infected sheep no later than 5 days thereafter (59). By doubling this time period and supported by the fact that the highest bacterial load was observed during the early stage of infection (60, 61), reliable results should be obtained with our sampling protocol 10 days after the last footbath. Besides the experimental study (59), no further evidence has been published that the within-flock prevalence does rise up to at least 20% during the 10-day waiting period under field conditions. Therefore, a subclinically infected flock may has been wrongly categorized as negative, because not every animal was sampled. Nevertheless, with our sample sizes and a test specificity and sensitivity of 100 and 90%, respectively, virulent *D. nodosus* strains would have been detected using random sampling at a within-flock prevalence of 10%. In addition, by performing risk-based sampling instead of a random selection of sheep, the chance of missing an *aprV2*-positiv sheep is lower. Therefore, we expect that even flocks with a within-flock prevalence below 10% would not have been wrongly classified as *aprV2*-negative. To allow for risk-based sampling without lame animals at the end of the study, the initial footrot score as well as any other claw problem were recorded as risk factors. Also, no (re-)outbreaks were reported in flocks without contact with other sheep for up to 325 days, although the summer of 2021 was one of the wettest in Switzerland,^6^ thus environmental conditions (wet and warm) would have been ideal for clinical signs to develop allowing for diagnosis by clinical evaluation of the flock by the farmers (19).

Prior to sampling, all sheep were scored as clinically healthy. However, on two farms, the first of two analyses confirmed *aprV2*-positive status. This finding is in agreement with other studies (23–25, 46) and demonstrates the superior diagnostic sensitivity of PCR analysis compared to clinical examination regarding the presence of virulent strains of *D. nodosus*.

The use of NSAIDs was found to be negatively associated with the recovery from advanced footrot lesions in our study. This result should be interpreted with caution, because no standard protocol was used to select animals for NSAID treatment. It might well be possible that the finding represents a reverse causality and only severe cases with a slower healing process were treated. In a previous study, NSAIDs did not decrease the time needed for recovery from lameness (53); nevertheless, their use has been recommended as gold standard therapy for individual sheep (62). The recommendation is supported by the fact that the response of chronically lame sheep to mechanically induced pain was only equal compared to healthy control animals after repeated treatment with an NSAID (63). Therefore, NSAIDs should always be recommended for lame sheep and be administered for animal welfare reasons. Further research to evaluate the effect of NSAIDs on healing of footrot lesions is warranted.

## Conclusions

Using the adapted treatment protocol from Greber et al. (25) with a non-carcinogenic and eco-friendly disinfectant, *aprV2*-positive strains of *D. nodosus* could not be detected by risk-based sampling 10 days after the last footbath in all 18 flocks included. Therefore, the candidate disinfectant is recommended for the planned nationwide footrot control program in Switzerland. The easily applicable management factor of implementing a prewash waterbath saves more than three disinfecting footbaths, reducing treatment duration considerably. In contrast, antibiotic therapy did not reduce the number of disinfecting footbaths, but individual and selective use of a long-acting oxytetracycline has a beneficial effect on the recovery of severely affected animals. To improve animal welfare, the selective use of antibiotics may be considered when planning the control program.

## Data Availability Statement

The original contributions presented in the study are included in the article/Supplementary Material, further inquiries can be directed to the corresponding author/s. Data can also be accessed through our community-supported repository: https://zenodo.org/record/6594511#.YpTCevlhpoy.link.

## Ethics Statement

The study was carried out in accordance with Swiss animal welfare legislation (approval number, BE57/19+; approval date, 29 July 2019) and approved by the involved cantonal animal welfare committees. Written informed consent was obtained from the owners for the participation of their animals in this study.

## Author Contributions

RS was responsible for data collection, statistical analysis, and writing the first draft of the manuscript. JB and SD supported the statistical analyses. SB assisted with data collection. MA and AS supervised the study and edited the manuscript. All authors contributed to the manuscript and approved the final version.

## Funding

The present study was funded by the Swiss Federal Food Safety and Veterinary Office (Bern, Switzerland; grant number 1.19.06), PROFUMA Spezialfutterwerke GmbH & Co. KG (Dormagen, Germany), and Dr. E. Graeub AG (Bern, Switzerland). Open access funding was provided by the University of Bern.

## Conflict of Interest

The authors declare that the research was conducted in the absence of any commercial or financial relationships that could be construed as a potential conflict of interest.

## Publisher's Note

All claims expressed in this article are solely those of the authors and do not necessarily represent those of their affiliated organizations, or those of the publisher, the editors and the reviewers. Any product that may be evaluated in this article, or claim that may be made by its manufacturer, is not guaranteed or endorsed by the publisher.

## Footnotes

^1^CHF1 = approx. UK£0.78, US$1.07, €0.91 at 20 September 2021.

^2^Federal Food Safety and Veterinary Office (FSVO). Available online at: https://www.blv.admin.ch/blv/de/home/tiere/tierseuchen/uebersicht-seuchen/alle-tierseuchen/moderhinke.html (accessed June 25, 2021).

^3^Institute of Veterinary Pharmacology and Toxicology, University of Zurich: Available online at: https://www.vetpharm.uzh.ch/perldocs/wirktxt.htm (accessed June 10, 2020).

^4^Ausvet. Available online at: https://epitools.ausvet.com.au/ (accessed June 23, 2020).

^5^R Core Team (2020). Available online at: https://www.R-project.org/ (accessed July 5, 2021).

^6^Federal Office of Meteorology and Climatology, MeteoSwiss. Available online at: https://www.meteoschweiz.admin.ch/home/aktuell/meteoschweiz-blog.subpage.html/de/data/blogs/2021/8/nasser-sommer.html (accessed February 4, 2022).

## Supplementary Material

Supplementary Figure 1 Schematic illustration of the risk-based sampling method.
