# Erratum: Field validation of a non-carcinogenic and eco-friendly disinfectant in a stand-in footbath for treatment of footrot associated with *aprV2*-positive strains of *Dichelobacter nodosus* in Swiss sheep flocks

**DOI:** 10.3389/fvets.2023.1185202

**Published:** 2023-03-27

**Authors:** Frontiers Production Office

**Affiliations:** Frontiers Media SA, Lausanne, Switzerland

**Keywords:** *Dichelobacter nodosus*, footrot, footbath, prewash waterbath, sheep

An omission to the funding section of the original article was made in error. The following sentence has been added: “Open access funding was provided by the University of Bern.”

The publisher apologizes for this error. The original article has been updated.

