# Supplementary figures and images for: Field Validation of a Non-carcinogenic and Eco-Friendly Disinfectant in a Stand-In Footbath for Treatment of Footrot Associated With aprV2-Positive Strains of Dichelobacter nodosus in Swiss Sheep Flocks
Source: Front Vet Sci. 2022 Jun 13;9:812638. doi: 10.3389/fvets.2022.812638 (PMC9237958; doi:10.3389/fvets.2022.812638)

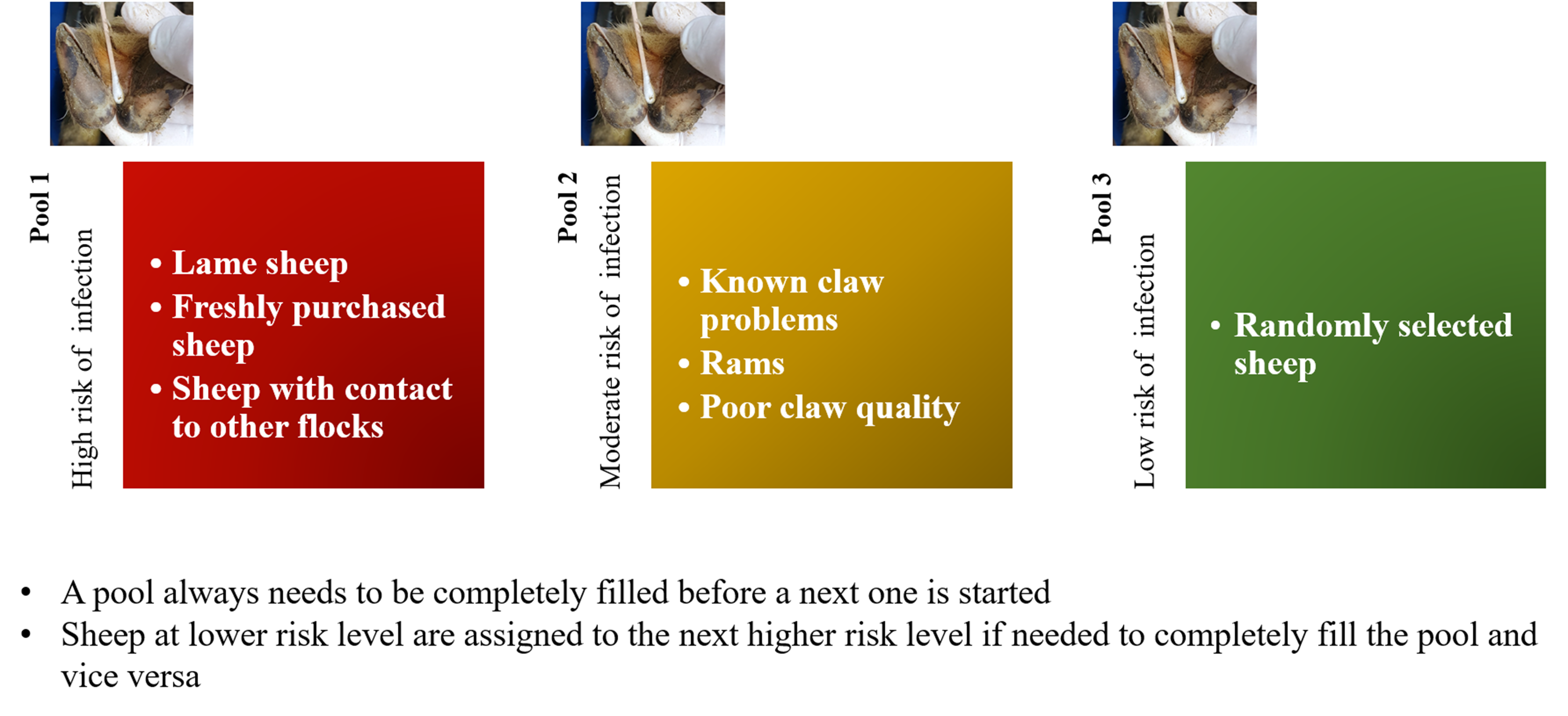

Supplement: Supplementary Figure 1 — Schematic illustration of the risk-based sampling method. [file Image_1.TIF]
