# Supplementary material for: Field Validation of a Non-carcinogenic and Eco-Friendly Disinfectant in a Stand-In Footbath for Treatment of Footrot Associated With aprV2-Positive Strains of Dichelobacter nodosus in Swiss Sheep Flocks
Source: Front Vet Sci. 2022 Jun 13;9:812638. doi: 10.3389/fvets.2022.812638 (PMC9237958; doi:10.3389/fvets.2022.812638)
Supplement: Supplementary file 2 [file Table_1.DOCX]

**Supplementary Table 1**

Definition of potential risk factors associated with individual animal recovery from footrot score ≥ 3 to score < 3 within two weeks (= four footbaths) in Swiss sheep flocks

| Potential risk factors | Statistical summary  (n=177 sheep) |
| --- | --- |
| **Flock factors (continuous)** |  |
| Flock size | 9 – 67  (median, 28) |
| Prevalence of footrot | 23 – 93%  (median, 49.5%) |
| Prevalence of advanced lesions^a^ | 11 – 71%  (median, 30.5%) |
| **Management factors** |  |
| Permanent access to a concrete outdoor paddock |  |
| Yes | 88 (49.7%) |
| No | 65 (36.7%) |
| Not applicable^b^ | 24 (13.6%) |
| Prewash waterbath prior to first footbath |  |
| Yes | 49 (27.7%) |
| No | 128 (72.3%) |
| Prewash waterbath prior to subsequent footbaths^c^ |  |
| Yes | 125 (70.6%) |
| No | 52 (29.4%) |
| Liming with calcium carbonate |  |
| Yes | 85 (48.0%) |
| No | 82 (46.3%) |
| NA^d^ | 10 (5.7%) |
| Mucking out and disinfecting at visit 1 |  |
| Yes | 63 (35.6%) |
| No | 104 (58.8%) |
| NA^d^ | 10 (5.7%) |
| **Animal factors** |  |
| Age category |  |
| Lambs (< 8 months) | 19 (10.7%) |
| Yearling (≥ 8 months, ≤ 12 months) | 13 (7.4%) |
| Ewe (female, > 12 months) | 138 (78.0%) |
| Ram (male, >12 months) | 7 (4.0%) |
| Sex |  |
| Male | 16 (9.0%) |
| Female | 161 (91.0%) |
| SWA^e^ |  |
| Yes | 27 (15.3%) |
| No | 150 (84.6%) |
| Other claw pathologies |  |
| Yes | 55 (31.1%) |
| No | 122 (68.9%) |
| Clinical footrot score |  |
| Score 3 | 91 (51.4%) |
| Score 4 | 81 (45.8%) |
| Score 5 | 5 (2.8%) |
| Number of clinically affected feet |  |
| 1 foot | 56 (31.6%) |
| > 1 foot | 121 (68.4%) |
| Number of severely affected feet^a^ |  |
| 1 foot | 98 (55.4%) |
| >1 foot | 79 (44.6%) |
| Antibiotic treatment^f^ |  |
| Yes | 106 (59.9%) |
| No | 71 (40.1%) |
| NSAID treatment^g^ |  |
| Yes | 54 (30.5%) |
| No | 123 (69.5%) |
| **Others** |  |
| Season at the start of the treatment |  |
| September – November | 52 (39.4%) |
| December – February | 114 (64.4%) |
| March - May | 11 (6.2%) |
| June - August | 0 |

^a^ score ≥ 3 according to BGK; ^b^ not applicable: sheep were kept on pasture; ^c^ on 7 farms, the prewash waterbath was not available from the beginning or could not be used because the court was needed for claw trimming; ^d^ NA: not available; ^e^ SWA: Swiss white alpine sheep; ^f^ one dose of a long-acting oxytetracycline (20mg/kg, IM); ^g^ NSAID: non-steroidal anti-inflammatory drug
